# Molecular-genetic and cytogenetic analyses of cotton chromosome introgression from Gossypium barbadense L. into the genome of G. hirsutum L. in BC2F1 hybrids

**DOI:** 10.18699/VJGB-23-110

**Published:** 2023-12

**Authors:** M.F. Sanamyan, Sh.U. Bobokhujayev, Sh.S. Abdukarimov, O.G. Silkova

**Affiliations:** National University of Uzbekistan named after Mirzo Ulugbek, Tashkent, Uzbekistan; National University of Uzbekistan named after Mirzo Ulugbek, Tashkent, Uzbekistan; Center of Genomics and Bioinformatics of the Academy of Sciences of the Republic of Uzbekistan, Tashkent, Uzbekistan; Institute of Cytology and Genetics of the Siberian Branch of the Russian Academy of Sciences, Novosibirsk, Russia

**Keywords:** cotton, Gossypium hirsutum, G. barbadense, monosomic lines, chromosome-substituted hybrids, molecular genetic analysis, хлопчатник, Gossypium hirsutum, G. barbadense, моносомные линии, хромосомно-замещенные гибриды, молекулярно-генетический анализ

## Abstract

Substitution lines of the cotton Gossypium hirsutum L. involving chromosomes of the tetraploid species G. barbadense L., G. tomentosum Nutt. ex Seem., and G. mustelinum Miers ex Watt. are a valuable source for breeding, increasing the genetic diversity of G. hirsutum. The substitution of certain G. hirsutum L. chromosomes with G. barbadense chromosomes affect fibre elongation, fibre yield, fibre strength, and micronaire. To increase the efficiency of creating lines, it is necessary to study the nature of the introgression of alien chromosomes into the G. hirsutum L. genome. As a result of molecular genetic analysis of BC2F1 hybrids obtained from crossing monosomic lines of the cotton G. hirsutum from the cytogenetic collection of Uzbekistan with monosomic backcross hybrids BC1F1 G. hirsutum × G. barbadense on the same chromosomes, genetic differences between the hybrids in the profile of chromosome-specific microsatellite SSR markers were found. The predominant introgression of chromosomes 4, 6 and 12 of the At-subgenome and 22 of the Dt-subgenome of G. barbadense was revealed, while chromosomes 2 and 7 of the At-subgenome and 18 of the Dt- subgenome of G. barbadense were characterized by elimination. Among them, chromosomes 7 of the At- subgenome and 18 of the Dt-subgenome of G. barbadense were eliminated in the first backcross generation. In this work, two lines, CS- B06 and CS-B07, from the American cytogenetic collection with a putative substitution involving chromosomes 6 and 7 of the At-subgenome were analysed. The presence of only polymorphic alleles from the species G. hirsutum and the absence of polymorphic alleles from the species G. barbadense were revealed, which showed the absence of substitution involving these chromosomes. BC2F1 hybrids with monosomy for both G. barbadense and G. hirsutum chromosomes were characterized by regular pairing of chromosomes and high meiotic indexes. However, many hybrids were characterized by a decrease in pollen fertility. Two hybrids with monosomy for chromosome 7 of the At-subgenome of G. hirsutum and chromosome 6 of the At-subgenome of G. barbadense had the greatest reduction in pollen viability (70.09 ± 1.57 and 75.00 ± 1.66 %, respectively). Thus, this work shows a specific feature in the introgression of individual chromosomes of the cotton species G. barbadense into the cotton G. hirsutum genome.

## Abstract (ru)

Линии хлопчатника Gossypium hirsutum L. с чужеродным замещением хромосом тетраплоидных ви- дов G. barbadense L., G. tomentosum Nutt. ex Seem., G. mustelinum Miers ex Watt. являются ценным источником для селекции, увеличивающим генетическое разнообразие G. hirsutum. Замещение определенных хромосом хлоп- чатника вида G. hirsutum L. хромосомами вида G. barbadense оказывает влияние на удлинение, выход и прочность волокна, микронейр. Для повышения эффективности процесса создания линий необходимо изучение характера интрогрессии чужеродных хромосом в геном G. hirsutum L. В результате молекулярно-генетического анализа гибридов BC2F1, полученных от скрещиваний моносомных линий хлопчатника G. hirsutum цитогенетической коллекции Узбекистана с моносомными беккроссными гибридами BC1F1 G. hirsutum × G. barbadense по одинако- вым хромосомам, обнаружены генетические различия по профилю хромосом-специфичных микросателлитных SSR-маркеров между гибридами. Выявлена преимущественная интрогрессия хромосом 4, 6, 12 At-субгенома и 22 Dt-субгенома G. barbadense, тогда как хромосомы 2, 7 At-субгенома и 18 Dt-субгенома G. barbadense характе- ризовались элиминацией, среди них хромосомы 7 At-субгенома и 18 Dt-субгенома G. barbadense элиминировали уже в первом беккроссном поколении. В настоящей работе проанализированы две линии, CS-B06 и CS-B07, аме- риканской цитогенетической коллекции с предполагаемым замещением по хромосомам 6 и 7 Аt-субгенома. Об- наружены присутствие только полиморфных аллелей вида G. hirsutum и отсутствие полиморфных аллелей вида G. barbadense, что показало отсутствие замещения по этим хромосомам. Гибриды BC2F1 с моносомией как по хромосомам G. barbadense, так и по хромосомам G. hirsutum характеризовались регулярной конъюгацией хромо- сом и высоким мейотическим индексом. Однако многие гибриды отличались снижением фертильности пыльцы. Два гибрида с моносомией по хромосоме 7 At-субгенома G. hirsutum и хромосоме 6 At-субгенома G. barbadense имели наибольшую редукцию в жизнеспособности пыльцы (70.09 ± 1.57 и 75.00 ± 1.66 % соответственно). Таким образом, в этой работе показана особенность в интрогрессии индивидуальных хромосом хлопчатника вида G. barbadense в геном хлопчатника G. hirsutum.

## Introduction

Currently, four species of cotton are grown commercially worldwide, of which two species, Gossypium herbaceum L. (A1-genome) and G. arboreum L. (A2-genome), are diploids, and the other two species, G. hirsutum L. (AD1-genome) and G. barbadense L. (AD2-genome), are tetraploids (Wendel et al., 2009). The cotton plant G. hirsutum is a major crop that accounts for more than 90 % of the world’s cotton crop (International Cotton Advisory Committee-ICAC-2019).

Global cotton consumption has shown a steady increase of 80 % between 1980/1981 and 2020/2021 (International Cotton Advisory Committee-ICAC-2021), requiring improvements in cotton yields and fibre quality. An increase in cotton yield was achieved through the creation of transgenic varieties, traditional selection, and intervarietal crossing. However, most of these varieties were obtained through selection from a narrow genotypic environment and adapted to certain soil and climatic conditions (International Cotton Advisory Committee-ICAC- 2021). Thus, today, there is a reduction in genetic diversity in cultivated cotton, which causes a decrease in fibre quality and increased vulnerability to stress factors due to the close relatedness of high-yielding varieties

Enrichment of the G. hirsutum genome with alleles of economically valuable genes from other cotton species is very important (Grover et al., 2022). For example, G. tomentosum is characterized by heat resistance, and G. mustelinum and G. stocksii are resistant to pests and diseases. It is known that fine-fibre cotton of the G. barbadense species is less productive and has less adaptability to growing conditions but has fibre properties that are significantly superior in quality (length, strength and fibre fineness) to the cultivated G. hirsutum varieties, although the latter is more productive. Given their complementary economically valuable traits, numerous attempts have been made to hybridize these two species through traditional breeding (Anwar et al., 2022). However, the interspecific hybrids had poor agronomically valuable traits, and the hybrids were characterized by limited recombination due to genomic incompatibility caused by large inversions on different chromosomes of the two subgenomes of the tetraploid species. Typically, F1 hybrids of G. hirsutum × G. barbadense are fertile, but the phenotypes of F2 and subsequent generations are biased towards one of their parents due to pollen sterility, suppression of crossing over, selective gene elimination and segregation failure (Zhang et al., 2014; Si et al., 2017; Fang et al., 2023).

Obtaining forms with chromosome substitution (CS) in various plant species allows for targeted introgression of specific chromosomes or arms of individual chromosomes, which represent a valuable source of new alleles of useful genes. Previously, such forms were created in many crops, which made it possible to improve some agronomic traits (Shchapova, Kravtsova, 1982; Silkova et al., 2006, 2007; Schneider et al., 2008; Tiwari et al., 2010; Rawat et al., 2011).

For a number of years, in cotton in the USA, research has been carried out to obtain lines with alien chromosome substitutions involving three tetraploid species (G. barbadense, G. tomentosum, G. mustelinum), and with the participation of the G. barbadense species, 20 lines with substitutions of individual chromosomes have already been obtained (Saha et al., 2006, 2013, 2015). The obtained lines made it possible to determine that the substitution of certain chromosomes of the cotton species G. hirsutum L. with chromosomes of the species G. barbadense L. (CS-B02, CS-B04, CS-B16, CS-B17, CSB22Lo, CS-B22sh, CS-B25) has an effect on fibre elongation, fibre yield, fibre strength, micronaire, etc., in comparison with the original lines TM-1 and Pima 3-79 (Saha et al., 2004). Such lines have been shown to be an important breeding source that increases the genetic diversity of G. hirsutum L. (Jenkins et al., 2006, 2007).

Previously, monosomic lines of the Cytogenetic Collection of Cotton of Uzbekistan (CCCU), created in the genotypic environment of the highly inbred line L-458 of the species G. hirsutum L. (Sanamyan et al., 2014), with identified monosomy on chromosomes 2, 4, 6, 7, 12 of the At-subgenome and 17, 18, 21, 22 of the Dt-subgenome, as well as two lines with monosomy on telocentrics 6 and 11 of the At-subgenome (Sanamyan et al., 2016a, b; Sanamyan, Bobokhujayev, 2019), were used in crossings with the Pima 3-79 line of the G. barbadense species, as well as in crossings with F1 hybrids, to obtain aneuploid hybrids BC1F1 and subsequently to create cotton lines with chromosome substitution. The work used double screening of hybrids at all stages of backcrossing using molecular genetic markers and cytogenetic analysis (Sanamyan et al., 2022). The first stage of the study consisted of a molecular genetic analysis of hybrid plants at the seedling stage to quickly identify aneuploid forms with or without chromosome substitutions or their arms. At the second stage, a cytogenetic analysis of meiosis in hybrids at the stages of metaphase I and telophase II was carried out, and pollen fertility when stained with acetocarmine was studied to confirm the monosomic status of backcross hybrid plants and identify their peculiarities in the behavior of chromosomes.

The purpose of this work was to conduct a molecular genetic and cytogenetic study of BC2F1 hybrids from crosses of monosomic cotton lines of the CCCU with monosomic backcross hybrids BC1F1 and to elucidate the features of introgression of individual chromosomes of the cotton species G. barbadense into the genome of the cotton species G. hirsutum. In the course of this work, at the seedling stage, using molecular genetic markers (SSR), aneuploid forms were identified among BC2F1 hybrids, in which the substitution of chromosomes 4, 6, and 12 of the At-subgenome and chromosome 22 of the Dt-subgenome and the elimination of chromosomes 2 and 7 of the At-subgenome and 18 Dt-subgenome with G. barbadense were confirmed. In aneuploids BC2F1, the behavior of individual chromosomes of G. hirsutum and G. barbadense in meiosis was studied, and the meiotic index and pollen fertility were assessed. The promise of using molecular genetic markers at the seedling stage for accelerated selection of plants with alien substitution of individual G. hirsutum/ G. barbadense chromosomes in the BC2F1 generation has been shown.

## Materials and methods

Plant material. Monosomic and monotelosomics lines of CCCU were created in a single genotypic environment of the highly inbred line L-458 of G. hirsutum, obtained by M.F. Abzalov and G.N. Fatkhullaeva as a result of long-term self-pollination (F20) based on variety 108-F. To create the collection, various methods were used to irradiate seeds and pollen, as well as the progeny of plants with translocations and desynapsis (Table 1) (Sanamyan, 2020). The Pima 3-79 line of the G. barbadense species is not sensitive to photoperiod and is highly homozygous, as it originates from a doubled haploid (Endrizzi et al., 1985). This line is the genetic standard for the species G. barbadense L. in the USA (Hulse-Kemp et al., 2015) and has therefore been used as the donor parent of the substituted chromosome (CS) or chromosome segments from G. barbadense, both in the USA and in Uzbekistan.

**Table 1.**
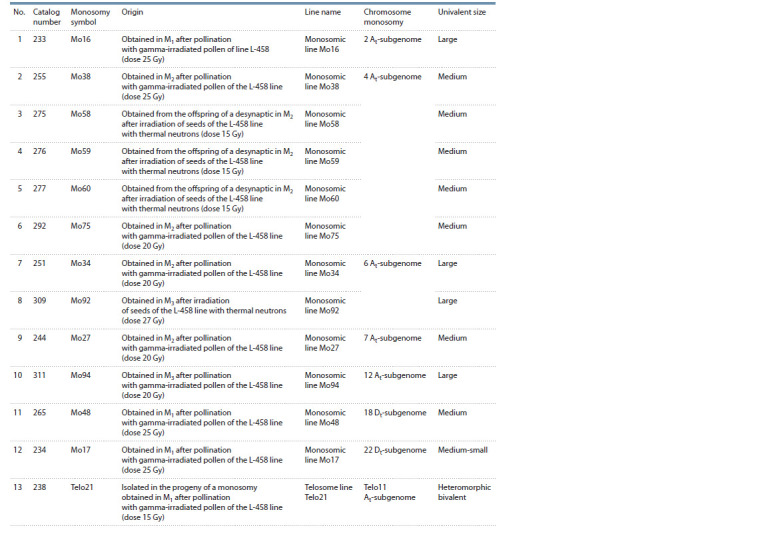
Monosomic and monotelosomal lines of cotton G. hirsutum L. cytogenetic collection of Uzbekistan

To obtain backcross hybrids BC2F1, monosomic lines on chromosomes 2, 4, 6, 7, and 12 of the At-subgenome and 18 and 22 of the Dt-subgenome were backcrossed with monosomic hybrids BC1F1(Mo × F1(Mo × Pima 3-79)), and a monotelosomic line lacking one of the arms of chromosome 11 was backcrossed with the monotelosomal hybrid BC1F1(Telo × F1(Telo × Pima 3-79)), in which monosomy and monotelosomy were on the same chromosomes as in the original aneuploids of G. hirsutum. All plants of the original lines and hybrids of different generations were kept year-round in the greenhouse of the National University of Uzbekistan.

Cytological tests. The behavior of chromosomes was studied in the pollen mother cells (PMCs) at the stage of metaphase I (MI) and tetrads of meiosis. For this, 2–3 mm buds in the ethyl-acetic acid mixture (7:3) were fixed. Then, the PMCs were painted with iron-acetocarmine. At temporary squashed slides at the MI stage, the nature of the pairing of chromosomes was taken into account. To analyse the stage of the tetrads, three buds were analysed from each plant, and the percentage of normal tetrads was calculated from their total number. To analyse the fertility of pollen, in the morning on the day of flowering, the opened flowers were collected, and temporary acetocarmine slides were prepared, which were laid in Petri’s cups and left in the refrigerator for a day to better paint the pollen grains. Then, 10 fields of vision from each flower were analysed.

All cytological observations were carried out using microscopes, AxioScopeA1, Laboval (Carl Zeiss, Germany) and Biomed (Leica, Switzerland) with an increase in lenses of 10x, 100x, binocular nozzle of 1.6x and GF 12.5 × 120 and a 10x eyepiece. Microphotography was performed using a Mikroskopkamera AxioCamERc5s digital camera. During exhibiting, the green filter 3C-11-3 was used. Statistical processing of the received data was carried out in accordance with B.A. Dospekhov (1985).

DNA extraction and genotyping. Genomic DNA was distinguished from samples of young leaves of cytogenetically identified backcross aneuploid hybrids BC2F1 and young seedlings of hybrid plants (BC2F1) by CTAB (Saha et al., 2015). Genomic DNA was checked using electrophoresis of 0.9 % agarose, and DNA was diluted in 15 μl to a working concentration using a control solution of HindIII-extensible DNA λ-fag (25 ng/ μl). The PCR amplification was carried out in 10 μl of the reaction mix containing 1.0 μl of 10-fold PCR buffer (with 25 mm MgCl2), 0.2 μl BSA, 0.08 μl dNTPs (25 mm), 0.2 μl of primers 0.1 μl Taq-polymerase, and 2 μl of DNA template. PCR runs were conducted with an initial DNA denaturation at 94 °C for 2 min, followed by 35 cycles of 94 °C (step 1) for 20 s, 55 °C (step 2) for 30 s and 72 °C (step 2, step 3) for 50 s. After 35 cycles, the extension temperature of 72 °C was held for 7 min. The PCR products were visualized in a 3.5 % high-resolution agarose gel, stained with bromide ethidium and photodocumented using an Alpha Imager gel documentation system (Innotech Inc., USA).

The pairs of primers to the codominant chromosome-specific SSR markers were synthesized in accordance with genetic mapping (Dellaporta et al., 1983; Gutiérrez et al., 2009; Saha et al., 2015; Reddy et al., 2020), which are listed in Supplementary Material 11. For each chromosome, an average of four loci polymorphic between L-458 (G. hirsutum) and Pima 3-79 (G. barbadense) were selected. The results of the electropherogram for the SSR were evaluated as a/b/h, where the a locus corresponded to the recipient L-458, the b locus corresponded to the Pima 3-79 donor line, and the h genotype corresponded to the BC1F1 and BC2F1 disomic hybrid. The elimination of the chromosomes of G. hirsutum in the monosomic hybrid of cotton BC1F1 and BC2F1 was determined by the lack of marker amplification by chromosomes of G. hirsutum (maternal) and the presence of only allele-specific products of PCR of G. barbadense (paternal) (Liu et al., 2000). For all types of substitutions of individual chromosomes as controls, DNA of chromosome-substitution lines of the American cytogenetic collection was used, with the exception of chromosome 2.


Supplementary Materials are available in the online version of the paper: https://vavilov.elpub.ru/jour/manager/files/Suppl_Sanamyan_Engl_27_8.pdf


## Results

Identification of substitutions of chromosomes G. barbadense/G. hirsutum in BC2F1 hybrids using chromosome-specific molecular genetic markers

According to the previously developed scheme (Sanamyan et al., 2022), the molecular genetic analysis of BC2F1 plants was carried out at the seedling stage before they were transplanted into the soil of the greenhouses to accelerate the release of monosomics through chromosomes of donor species to separate their molecular markers from plants with chromosomes of the recipient species. Since most of the monosomics were identified earlier, only two crossing variants, BC2F1(Mo16 × BC1F1(9237) and BC2F1(Mo38 × BC1F1(92510)), were analysed at the seedling stage

The results of the analysis were discovered by five monosomics (211, 212, 214, 217 and 221, where the numbers indicate sowing plant numbers) in two families (21n and 22n, where the numbers indicate the sowing numbers of the families), and the letter “n” for a different number of plants in the BC2F1 (Mo16 × BC1F1 (9237)) variant, where there was supposed to be a substitution of chromosome 2 of the At-subgenome. These plants were characterized by the presence of chromosome- specific alleles only from the L-458 line G. hirsutum, while the G. barbadense alleles were absent. Since earlier the chromosome-specific SSR markers BNL834, BNL3971, TMB0471, and JESPR179 had been localized on chromosome 2 of the At-subgenome of cotton (Gutiérrez et al., 2009; Lacape et al., 2009) (see Supplementary Materials 1–3), the data obtained indicated the lack of chromosome 2 substitution in all five backcross seedlings in BC2F1(Mo16 × BC1F19237), which was a negative result of this study, as it made it necessary to obtain further new hybrid background seeds and study the new BC2F1 hybrid offspring.

**Fig. 1.**
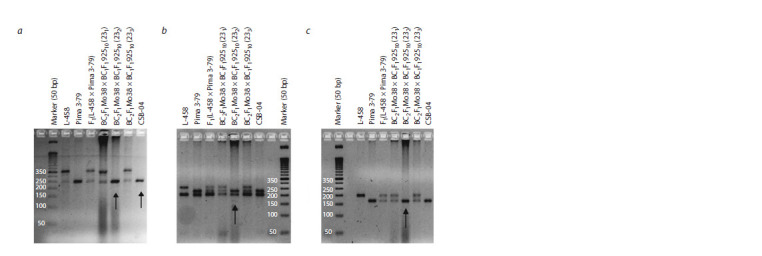
Electrophoregram of the DNA amplicons of SSR markers in hybrid seedlings of BC2F1(Mo38 × BC1F192510) according to chromosome 4 of the At-subgenome: a – Gh107; b – Gh117; c – TMB0809

One seedling (232) with substitution of chromosome 4 was found in the BC2F1(Mo38 × BC1F192510). This hybrid was characterized by the presence of alleles only from G. barbadense, which was revealed upon receipt of PCR products as a result of amplification with four chromosome-specific SSR markers: BNL2572, GH107, GH117, and TMB0809 (Hoffman et al., 2007; Gutiérrez et al., 2009) (see Supplementary Materials 1, 2; Fig. 1).

Confirmation of chromosomal substitutions in the other 10 variants was carried out in previously cytogenetically studied BC2F1 monosomic hybrids. Analysis of monosomics with a putative substitution in chromosome 4 showed amplification of five allele-specific PCR products of SSR markers TMB0809, Gh107, Gh117, CIR249, JESPR234 only for G. barbadense in monosomic (5301) from the variant of BC2F1(Mo58 × BC1F11151), in two monosomics (2841 and 28411) in BC2F1(Mo59 × BC1F110414), in monosomic (4943) from BC2F1(Mo60 × BC1F11175), and in monosomic (4961) in BC2F1(Mo75 × BC1F12982) (see Supplementary Materials 1, 4, 5), which confirmed the substitution of the chromosomes in them.

**Fig. 2.**
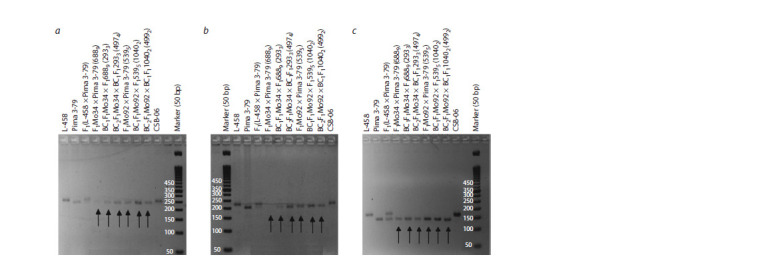
Electrophoregram of SSR-marker DNA amplicons in hybrid monosomic plants BC2F1(Mo34 × BC1F1(2933)) and BC2F1(Mo92 × F1BC1(10402)) according to chromosome 6 of the At-subgenome of cotton: a – TMB0853; b – TMB1538; c – Gh082.

Analysis of monosomic (4974) in the BC2F1(Mo34 × BC1F1 (2933)) variant and monosomic (4992) in the BC2F1(Mo92 × BC1F1(10402)) variant with a putative substitution of chromosome 6 revealed alleles only from G. barbadense, while alleles of the G. hirsutum species were absent, based on the localization of 11 chromosome-specific SSR markers BNL1440, BNL3650, BNL2884, BNL1064, BNL3359, TMB1277, TMB0154, TMB0853, TMB1538, Gh039, and Gh082 (Gutiérrez et al., 2009) ((see Supplementary Materials 1, 5; Fig. 2), substitution of these chromosomes was confirmed.

The molecular genetic analysis of two monosomics (50011 and 50012) from BC2F1(Mo27 × BC1F1(1112)) defined only the alleles of the L-458 G. hirsutum line, while alleles of the G. barbadense species were absent. Before four chromosomespecific SSR markers, BNL1694, Gh146, TMB0180, and TMB0561, were localized on chromosome 7 of the At-subgenome (Hoffman et al., 2007; Guo et al., 2008; Gutiérrez et al., 2009; Saha et al., 2015) (see Supplementary Mate-rials 5, 6), the data obtained indicated the lack of substitution of chromosome 7 in these two monosomics

It must be emphasized that the substituted CS-B06 and CS-B07 lines of the American cytogenetic collection that served as control in our study were characterized by the lack of substitution of chromosomes 6 and 7 of cotton, since only those from the species of G. hirsutum were present, while those from the species G. barbadense were absent, as can be clearly seen in Fig. 2 and Supplementary Material 6, respectively. However, all other controls corresponded to the substitutions of the chromosomes by which the study was conducted.

In two monosomics (5054 and 5062) from the BC2F1 variant (Mo94 × BC1F12991), only chromosome 12 of the At-subgenome of G. barbadense was identified according to the PCR of the amplification of chromosome-specific SSR markers- BNL3261 and BNL3835 (Gutiérrez et al., 2009) (see Supplementary Materials 5, 7).

Analysis of monosomic (28614) from the combination of BC2F1(Mo48 × BC1F111420) showed only alleles of chromosome 18 from G. hirsutum, while alleles of the species G. bar-badense were absent. Since eight previously reported chromosome- specific SSR markers, namely, BNL193, BNL2544, BNL3280, BNL3479, CIR216, Gh142, TMB0114, and TMB1603, were localized on chromosome 18 of the Dt-subgenome (Reddy et al., 2020) (see Supplementary Materials 5, 8), the data indicated the lack of substitution of this chromosome.

The molecular-genetic SSR analysis of monosomic (2881) from BC2F1(Mo17 × BC1F11101) showed the presence of only the allele from G. barbadense, while the allele of the G. hirsutum species was not found based on the localization of the chromosome-specific SSR marker BNL673. Since this marker was previously localized on chromosome 22 of the Dt-subgenome (Gutiérrez et al., 2009), the substitution of chromosome 22 was confirmed in the studied monosomic (see Supplementary Materials 5, 9).

The molecular genetic analysis of two telocentrics (7902 and 7911) from BC2F1(Telo21 × BC1F1(2921)) showed conflicting data, possibly due to the localization of markers on different arms of chromosome 11. Therefore, the study of these monotelocentrics will be continued with the help of labelled primers since they show their more accurate localization.

Study of meiosis in BC2F1 hybrids with identified univalents

Analysis of the pairing of chromosomes at the MI meiosis stage revealed aneuploid plants in 12 variants of hybrid offspring obtained from the crosses of monosomic lines of the G. hirsutum species of the CCCU with monosomics of BC1F1. Therefore, two monosomics were isolated in each of the three backcrosses (with the participation of lines Mo59, Mo27 and Mo94), and one monosomic was allocated in each of the remaining nine backcross variants (with the participation of Mo16, Mo38, Mo58, Mo60, Mo75, Mo34, Mo92, Mo48 and Mo17) (Supplementary Material 10). Unfortunately, we were not able to continue research with four lines (Mo31, Mo56, Mo42 and Telo12), which were studied in the first backcross generation, due to the lack of setting of hybrid bolls

Analysis of metaphase I meiosis in 15 BC2F1 monosomics, where four monosomics (211, 50011 and 50012, 28614) of three crossing variants with univalent chromosomes of G. hirsutum (2, 7 and 18) and 11 other monosomics of eight other variants with univalent chromosomes of G. barbadense (4, 6 and 12) found that the plants were characterized by a modal for monosomics of cotton pairing of chromosomes with 25 bivalents and one univalent (Table 2). One monosomic variant (2881) from F1BC2(Mo17 × F1BC11101) with the substitution of chromosome of 22 of the Dt-subgenome was distinguished by the presence of additional univalents (1.94 ± 0.19 per cell), which could lead to the appearance of nullisomic gametes.

**Table 2.**
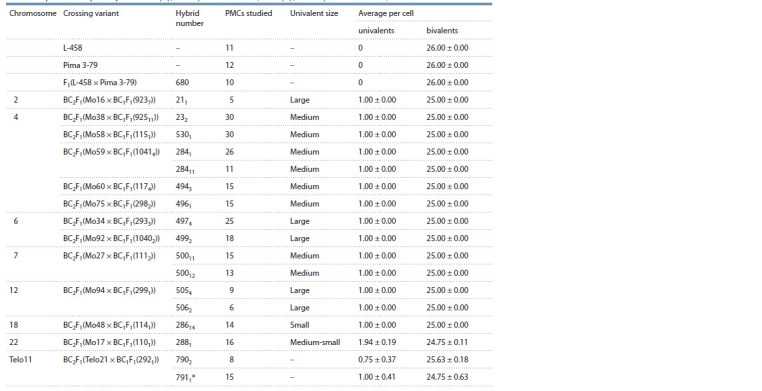
Pairing of chromosomes at the stage of metaphase I meiosis in BC2F1, hybrids obtained from crossing recurrent parents with interspecific aneuploid hybrids of BC1F1(Mo × F1Mo × Pima 3-79) or BC1F1(Telo × F1Telo × Pima 3-79) * 0.25 ± 0.25 quadrivalents on average per cell in monotelosomal plant 7911.

In monotelosomics (7902 and 7911) in the BC2F1 variant (Telo21 × BC1F1(2921)), paired univalents (0.75 ± 0.37 and 1.00 ± 0.41 per cell, respectively), along with heteromorphic bivalents, were found in separate PMCs. One monotelosomic (7911) also formed one quadrivalent (0.25 ± 0.25 per cell) (see Table 2).

**Fig. 3.**
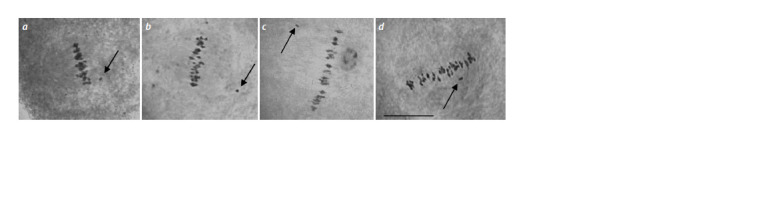
Chromosome configurations in metaphase I of meiosis in hybrid BC2F1 plants obtained from crossing monosomic lines with interspecific monosomic hybrids BC1F1(25II+1I). a – BC2F1(Мо58 × BC1F1(1151)) (5301); b – BC2F1(Mo59 × BC1F1(10414)) (2841); c – BC2F1(Мо60 × BC1F1(1175)) (4943) (25II+1I) with chromosome 4 of G. barbadense; d – BC2F1(Mo34 × BC1F1(2933)) (4974) with chromosome 6 of G. barbadense. Here and in Fig. 4: Arrows indicate univalents. Scale bar = 10 μm.

**Fig. 4.**
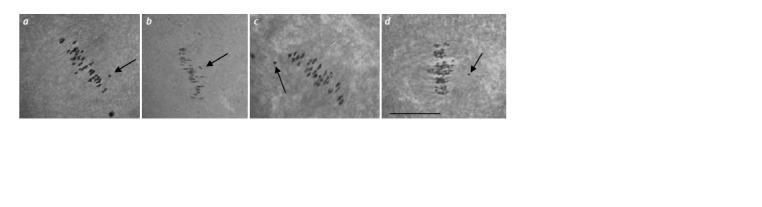
Chromosome configurations in metaphase I of meiosis in hybrid BC2F1 plants obtained from crossing monosomic lines with interspecific monosomic hybrids BC1F1(25II+1I). a – BC2F1(Mo27 × BC1F1(1112)) (50012) with chromosome 7 of G. hirsutum; b – BC2F1(Mo94 × BC1F1(2991)) (5054) with chromosome 12 of G. barbadense; c – BC2F1(Mo48 × BC1F1(11420)) (28614) with chromosome 18 of G. hirsutum; d – BC2F1(Mo17 × BC1F1(1101)) (2881) with chromosome 22 of G. barbadense.

Analysis of the size of univalents in monosomic BC2F1 revealed a large size of chromosome 2 of G. hirsutum in one family BC2F1(Mo16 × BC1F1(9237)), chromosome 6 of G. barbadense in two families, BC2F1(Mo34 × BC1F1(2933)) and BC2F1(Mo92 × BC1F1(10402)) (see Fig. 3, d ), and chromosome 12 of G. barbadense in one family, BC2F1(Mo94 × BC1F1(2991)) (see Fig. 4, b). BC2F1 monosomics in five families with chromosome 4 of G. barbadense BC2F1(Mo38 × BC1F1(92511)), BC2F1(Mo58 × BC1F1(1151)), BC2F1(Mo59 × BC1F1(10414)), BC2F1(Mo60 × BC1F1(1174)) and BC2F1 (Mo75 × BC1F1(2982)) (see Fig. 3, a–c), as well as with chromosome 7 of G. hirsutum BC2F1(Mo27 × BC1F1(1112)) (Fig. 4, a), had a medium size of univalents, which confirmed that they belong to the At-subgenome

A study of the size of the univalent in the plant (2881) variant of crosses of BC2F1(Mo17 × BC1F1(1101)) with chromosome 22 of G. barbadense revealed a medium-small size of the univalent (see Fig. 4, d ); in a plant of another variant, BC2F1(Mo48 × BC1F1(1141)) with chromosome 18 of G. hirsutum, it was small in size, which further confirmed that the chromosomes belong to the Dt-subgenome (Fig. 4, c).

**Fig. 5.**
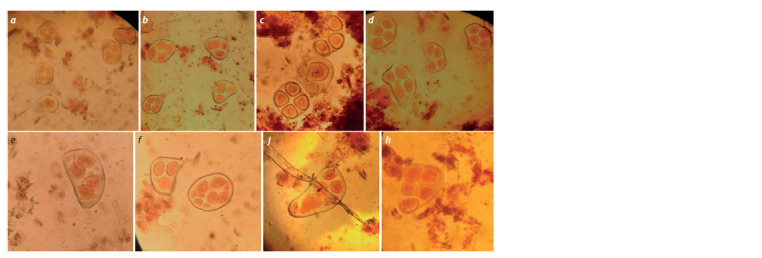
Sporades in the monosomic hybrid plant BC2F1(Mo34 × BC1F1(2933)) (4974): a – monad with micronuclei; b – triads and tetrads; c – monad with micronuclei and tetrads; d–f – tetrads with micronuclei; g – pentad with micronuclei; h – pentad.

Most BC2F1 monosomics showed a high meiotic index, which indicated that their univalent chromosomes underwent regular segregation (Supplementary Material 11). However, one monosomic variant, BC2F1(Mo34 × BC1F1(2933)), with a substitution of chromosome 6, demonstrated a decrease in the meiotic index (83.66 ± 0.62) and an increase in the number of tetrads with micronuclei (9.23 ± 0.77 %) (Fig. 5). This indicated disturbances in the divergence of chromosomes and the formation of unbalanced gametes, which could lead to “a univalent shift” in the offspring. Five monosomics in the BC2F1(Mo60 × BC1F1(1174)), BC2F1(Mo92 × BC1F1(10402)), BC2F1(Mo94 × BC1F1(2991)) and BC2F1(Mo17 × BC1F1(1101)) variants also showed a slight increase in the number of tetrads with micronuclei (from 1.22 ± 0.43 up to 1.84 ± 0.37 %), which could also lead to the same consequences (Supplementary Material 12, see Fig. 5). Similar to chromosome pairing, the meiotic index showed no significant differences between backcrossed monosomics with or without single chromosome substitutions

Two monotelosomics from the BC2F1 family (Telo21 × BC1F1(2921)) showed an increase in the percentage of tetrads with micronuclei from 2.17 ± 0.30 % (7911) to 2.32 ± 0.30 % (7902), which could be a consequence of a disturbance in the disjunction of the telocentric and the formation of unbalanced gametes in these hybrids (see Supplementary Material 12).

**Fig. 6.**
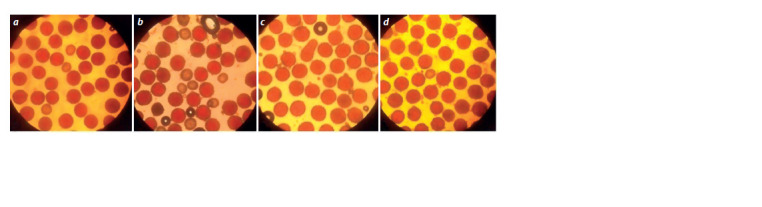
Fertile (colored) and sterile (uncolored) pollen in monosomic hybrids BC2F1 obtained from crossing monosomic lines with monosomic hybrids BC1F1(Mo × F1Mo × Pima 3-79): a, b – ВС2F1(Мо75 × BC1F12982) (4961); c, d – F1BC2(Mo34 × F1BC12933) (4974).

Pollen viability was assessed in BC2F1 monosomics using acetocarmine staining. Most of them showed high pollen viability (from 90.22 ± 1.31 to 96.15 ± 0.69 %), similar to line L-458 (90.92 ± 1.15 %) (Supplementary Material 13). Specifically, two monosomics (50012 and 4992) in two variants of crosses, BC2F1(Mo27 × BC1F1(1112)) and BC2F1(Mo92 × BC1F1(10402)) with chromosome 7 of G. hirsutum and with chromosome 6 of G. barbadense, had the greatest reduction in pollen viability (70.09 ± 1.57 and 75.00 ± 1.66 %, respectively) (Fig. 6), but four monosomics showed a slight reduction in pollen viability (from 83.20 ± 2.39 to 87.50 ± 1.95 %). However, in one variant, BC2F1(Mo59 × BC1F1(10414)), two monosomics were characterized by differences in pollen viability of more than 17 %, and in another variant, BC2F1 (Mo27 × BC1F1(1112)), these differences were more than 20 %

## Discussion

In recent years, a comprehensive analysis of alien addition and alien substitution lines, including morpho-biological, genetic, cytogenetic and molecular genetic methods, has proven itself (Schneider, 2010; Tiwari et al., 2010; Rawat et al., 2011; Garg et al., 2016).

An integrated approach using differential C-staining, fluorescence in situ hybridization (FISH) and gliadin analysis in analyses of introgression lines of T. aestivum × Ae. columnaris allowed to identify substitutions, addition chromosomes or fragments of individual chromosomes in 15 lines, while in five lines, the presence of alien genetic material was not detected (Shishkina et al., 2017). In a study of introgression lines obtained from backcrosses with bread wheat varieties of the synthetic form RS7 (BBAAUS), using C-staining, FISH, and DNA markers, lines with substitution of wheat chromosomes and with chromosome rearrangements were found; however, two lines were characterized by the absence of alien introgressions (Davoyan et al., 2019). It has become obvious that in studies of the genomic composition of alien substituted forms, it is extremely necessary to use a complex of cytological and molecular genetic methods.

In cotton, studies using SSR markers and genomic in situ hybridization (GISH) have also been initiated, which allowed the isolation of five monosomic alien addition lines (MAALs) in the backcross progeny of a pentaploid obtained from crosses of the species G. hirsutum with the Australian diploid species G. australe F. Muell. (Sarr et al., 2011). The use of BAC-FISH probes in five diploid cotton species allowed to successfully identify individual chromosomes and map 45S and 5S rDNA to specific chromosomes of five species (Gan et al., 2012). Comparison of the cytogenetic map of chromosome 1 of the species G. herbaceum L., constructed using BAC-FISH, with the genetic maps of chromosome 1 of the species G. hirsutum, G. arboreum, and G. raimondii showed that most of the identified BAC clones are located in the same order on different maps, with the exception of three markers indicating chromosome rearrangements (Cui et al., 2015). Unfortunately, such complex analysis methods have not yet been used to study chromosome substitution lines.

Modern genotypes of cultivated cotton are characterized by restriction of alleles for beneficial traits due to monophyletic origin and the formation of a “genetic bottleneck” that arose during domestication from a common ancestor and crosses between the same genotypes of elite forms (Saha et al., 2018). This has stimulated the search for genetic diversity among different cotton species

The creation of 17 substituted cotton lines (CS-B), where each homologous pair of chromosomes or chromosomal arms of the species G. hirsutum (TM-1) was substituted by a homologous chromosome or arm of the species G. barbadense (Pima 3-79) (Stelly et al., 2005), made it possible to associate the most important traits of fibre quality with a single chromosome or its arm (Saha et al., 2004; Jenkins et al., 2006), to begin the introgression of favorable genes for the improvement of cultivated cotton (Jenkins et al., 2006, 2007) and to study chromosomal effects on agronomic traits (fibre yield, boll weight, raw cotton yield) and data processing using a genetic model (ADAA) (Saha et al., 2010).

Later, some of these cotton lines did not receive moleculargenetic confirmation (Gutiérrez et al., 2009; Saha et al., 2015; Ulloa et al., 2016). In recent work, chromosome-specific markers (SSRs) were used in a MAGIC population created by crossing 18 CS-B lines with three Upland cotton cultivars. Ultimately, the same five lines (CS-B05sh, CS-B06, CS-B07, CS-B12sh and CS-B15sh) that were listed in previous articles contained “little or no introgression of the whole chromosome or chromosome region” (Fang et al., 2023). Only 13 CS-B lines contained “significant introgression” from the G. barbadense species, and the reasons for the lack of molecular-genetic confirmation in some chromosome substitution cotton lines remain unclear.

When creating cotton lines with G. barbadense/G. hirsutum chromosome substitution, the selection of plants with the needed genotype was accelerated thanks to molecular-genetic testing of backcross plants at the seedling stage (Sanamyan et al., 2022). This also contributed to the continuation of backcrossing of only those hybrid forms that had the desired genotype. This rapid selection of plants with the desired genotype underscored the advantages of using molecular markers (SSRs) in such studies

In this work, using chromosome-specific SSR markers in the BC2F1(Mo16 × BC1F1(9237)) variant in six seedlings with monosomy, the elimination of chromosome 2 of the G. barbadense At-subgenome and the presence of chromosome 2 of the G. hirsutum At-subgenome were detected, while in one seedling of another family BC2F1(Mo38 × BC1F1(92510)) chromosome 4 of the At-subgenome of G. barbadense was revealed, which indicates chromosome substitution in this plant. Confirmation of chromosome substitutions carried out by molecular genetic analysis in previously cytogenetically identified monosomic BC2F1 hybrids was established only on chromosomes 4, 6, and 12 of the At-subgenome and chromosome 22 of the Dt-subgenome of cotton in eight variants, while in two variants, BC2F1(Mo27 × BC1F1(1112)) and BC2F1(Mo48 × BC1F1(11420)), the absence of substitution of chromosome 7 of the At-subgenome and 18 of the Dt-subgenome was revealed. Consequently, the lack of elimination of chromosome 4 of the At-subgenome of G. barbadense in the five studied backcross variants (involving lines Mo38, Mo58, Mo59, Mo60 and Mo75) indirectly indicates its preferential transmission through gametes, while the elimination of chromosomes 7 of the At-subgenome and 18 of the Dt-subgenome of G. barbadense already in the first backcross generation indicates their non-competitiveness in comparison with homeologues of G. hirsutum.

It must be emphasized that the presence of PCR products obtained as a result of amplification only with chromosomespecific SSR markers for chromosomes 6 and 7 of the At-subgenome of G. hirsutum in two lines (CS-B06 and CS-B07) of the American cytogenetic collection, which served as controls in our study, was a new confirmation of the incorrect determination of the substitution of chromosomes 6 and 7 of the At-subgenome, which had previously been emphasized by other researchers (Gutiérrez et al., 2009; Ulloa et al., 2016). In this regard, elucidating the reasons for the lack of introgression of donor chromosome 2 of the At-subgenome of G. barbadense during the backcrossing of hybrids is of great interest for future research.

To date, the reasons for the elimination of donor chromosomes in backcross hybrids remain unclear; however, it is known that in wheat-rye lines, the frequency of introgression of an alien chromosome depends both on the genotype of the line and on the genotype of the variety used in the crossing (Krasilova et al., 2011). Analysis of introgression lines of hybrid wheat with Aegilops columnaris Zhuk. showed that introgression processes depend on the parental wheat genotype and the level of divergence of homeologous chromosomes of the parent species (Badaeva et al., 2018). The chromosomes of those species that are taxonomically diverged from bread wheat to a greater extent are characterized by a low compensatory ability, which could be caused by structural rearrangements. Since no studies have yet been carried out in cotton to elucidate the factors influencing the frequency of introgression of an alien chromosome, studies of introgressive lines of wheat can contribute to the understanding of similar processes in other plant species.

All of the above can further clarify the processes causing the elimination of the donor chromosome of G. barbadense to occur during backcrossing in some types of crosses, but today it is known that the chromosomes of the Dt-subgenome of cotton have fewer small inversions than the chromosomes of the At-subgenome (Chen et al., 2020). In addition, tetraploid cotton has two reciprocal translocations, Chr.4/Chr.5 and Chr.2/Chr.3, which arose after polyploidization, and were confirmed by the presence of homologous loci (Wang et al., 2016). Additionally, inversions were found on many chromosomes, excluding chromosomes Chr.1, Chr.6, Chr.10, Chr.11, Chr.14, Chr.16, Chr.21, Chr.22 and Chr.24. All of the above structural changes in the chromosomes of tetraploid cotton could contribute to the difficulties that arose during the introgression of homeologous chromosomes.

A comparative analysis of chromosome pairing in backcross monosomics of different crossing variants revealed only single monosomics with additional univalents BC2F1(Mo17 × BC1F1(1101)), which theoretically could lead to a “univalent shift” in the offspring. However, as the study showed, the elimination of the G. barbadense chromosome during the process of backcrossing was observed in the offspring of other backcrossing hybrids with modal pairing of chromosomes, which indicated the existence of a mechanism for eliminating an alien chromosome, independent of the pairing of chromosomes and their subsequent disjunction.

However, it was expected that in one variant of crosses BC2F1(Mo34 × BC1F1(2933)) in hybrid monosomic (4974) with modal chromosome pairing, any disturbances in the genotype of the offspring could occur due to the formation of partially unbalanced gametes due to a reduced meiotic index (83.66 ± 0.62) and an increased percentage of tetrads with micronuclei (up to 9.23 ± 0.77 %). Therefore, the discovery in the next backcross generation BC3F1(Mo34 × BC2F14974) of five seedlings without substitution of chromosome 6 of the At-subgenome of cotton was predictable and indicated the exclusivity of the predicted event (Sanamyan, unpublished).

Assessment of pollen fertility after staining with acetocarmine in aneuploid backcross cotton plants revealed a decrease in different variants, which indicated the abortion of nullisomal gametes. Often, in the same crossings, monosomic hybrids were characterized by differences in the number of viable pollen. On the other hand, it is not possible to explain differences in the genotypes of monosomic hybrids only by differences in pollen fertility. It was previously shown that the assessment of pollen fertility after staining with acetocarmine in the progeny of monosomic cotton plants is not entirely convincing as a method for separating monosomic and disomic plants due to the abortion of unbalanced microspores in early development (Brown, Endrizzi, 1964). This assessment indicates the structural variability of genomes of interspecific monosomic hybrids with and without alien chromosome substitution. This variability at the level of chromosome behavior in the first division of meiosis is not detected using routine staining methods, but at the level of pollen viability, it is clearly visible.

## Conclusion

This work shows a peculiarity in the introgression of individual chromosomes of the cotton plant G. barbadense into the genome of the cotton plant G. hirsutum. Chromosomes 4, 6, and 12 of the At-subgenome and 22 of the Dt-subgenome of G. barbadense showed predominant introgression; BC2F1 hybrids with monosomic G. barbadense/G. hirsutum substitution were obtained on these chromosomes. Chromosomes 2, 7 of the At-subgenome and 18 of the Dt-subgenome of G. barbadense were characterized by elimination; among them, chromosomes 7 of the At-subgenome and 18 of the Dt-subgenome of G. barbadense were eliminated in the first backcross generation.

## Conflict of interest

The authors declare no conflict of interest.
